# Linoleic Acid-Induced Mitochondrial Ca^2+^ Efflux Causes Peroxynitrite Generation and Protein Nitrotyrosylation

**DOI:** 10.1371/journal.pone.0006048

**Published:** 2009-06-26

**Authors:** Hong-Mei Zhang, Howard Dang, Chih-Ko Yeh, Bin-Xian Zhang

**Affiliations:** 1 Department of Medicine, University of Texas Health Science Center at San Antonio, San Antonio, Texas, United States of America; 2 Department of Community Dentistry, University of Texas Health Science Center at San Antonio, San Antonio, Texas, United States of America; 3 Department of Dental Diagnostic Science, University of Texas Health Science Center at San Antonio, San Antonio, Texas, United States of America; 4 Geriatric Research, Education and Clinical Center, South Texas Veterans Health Care System, Audie L. Murphy Division, San Antonio, Texas, United States of America; L' Istituto di Biomedicina ed Immunologia Molecolare, Consiglio Nazionale delle Ricerche, Italy

## Abstract

It is well known that excessive non-esterified fatty acids in diabetes contribute to the pathogenesis of renal complications although the mechanism remains elusive. Enhanced oxidative stress has been hypothesized as a unified factor contributing to diabetic complications and increased protein nitrotyrosylation has been reported in the kidneys of diabetic patients. In the current manuscript we described that linoleic acid (LA) caused mitochondrial Ca^2+^ efflux and peroxynitrite production, along with increased nitrotyrosine levels of cellular proteins in primary human mesangial cells. The peroxynitrite production by LA was found to depend on mitochondrial Ca^2+^ efflux. Downregulation of hsp90β1, which has been previously shown to be essential for polyunsaturated fatty acid-induced mitochondrial Ca^2+^ efflux, significantly diminished LA-responsive mitochondrial Ca^2+^ efflux and the coupled peroxynitrite generation, implicating a critical role of hsp90β1 in the LA responses. Our results further demonstrated that mitochondrial complexes I and III were directly involved in the LA-induced peroxynitrite generation. Using the well established type 2 diabetic animal model db/db mice, we observed a dramatically enhanced LA responsive mitochondrial Ca^2+^ efflux and protein nitrotyrosylation in the kidney. Our study thus demonstrates a cause-effect relationship between LA and peroxynitrite or protein nitrotyrosylation and provides a novel mechanism for lipid-induced nephropathy in diabetes.

## Introduction

Elevated non-esterified fatty acids (NEFA) have been reported in patients and animal models of type 2 diabetes and shown to be associated with increased reactive oxygen species (ROS) production from mitochondria [1], [2] and nonmitochondrial sources [3]. Theoretically, excessive NEFA may influence mitochondrial ROS production by affecting the regeneration of reduced glutathione, proton gradients and electron transport [1]. Ionic Ca^2+^ in the mitochondria ([Ca^2+^]_m_) regulates not only substrate oxidation and ATP production [4], [5], but also ROS generation [6], which has been demonstrated to be a major contributing factor to the pathogenesis of diabetic complications including diabetic nephropathy [7]. Under normal physiological conditions, Ca^2+^ diminishes ROS production from both complexes I and III [6]. Whether excessive NEFA contribute to the massive ROS generation in diabetic nephropathy by alterating [Ca^2+^]_m_ is currently unknown.

Superoxide interacts with nitric oxide (NO) to form peroxynitrite, a strong oxidant that attacks various biomolecules and causes functional defects in cells and tissues [8]. Peroxynitrite interacts with and modifies multiple proteins and enzymes at tyrosine residues to form nitrotyrosine. Protein nitrotyrosylation may either up- or down-regulate activities of cellular pathways in a target-dependent manner [8]–[10]. Increased formation of nitrotyrosine has been demonstrated in diabetic kidneys [11] and may be associated with the pathogenesis of nephropathy.

In advanced diabetic nephropathy, mesangial cells are major contributors to glomerular mesangial matrix expansion and capillary basement membrane thickening with increased expression of the extracellular matrix components collagen IV, fibronectin, and laminin [12]. Human mesangial (HM) cells grown in high glucose show largely increased mitochondrial membrane potential and ROS production [13]. Elevated ROS generation may activate NF-κB and contribute to increased extracellular matrix accumulation.

NEFA may influence mitochondrial function by altering gene expression [14], metabolism [15], and/or mitochondrial Ca^2+^ homeostasis [4], [16]. We have recently reported that polyunsaturated fatty acids (PUFA) induce Ca^2+^ efflux from mitochondria [16], [17], an action that may disrupt mitochondrial function and influence ROS generation. It has yet to be determined whether PUFA-induced mitochondrial Ca^2+^ efflux (PIMCE) links to ROS generation. In the current study, we demonstrated that linoleic acid (LA, an 18∶2 n-6 PUFA)-responsive mitochondrial Ca^2+^ efflux caused peroxynitrite generation in HM cells. Our results also showed increased nitrotyrosylation of proteins in LA-treated HM cells and in the kidney of db/db diabetic mice, which may in turn impair kidney function and contribute to the pathogenesis of diabetic renal complications.

## Results

### LA caused [Ca^2+^]_i_ mobilization, peroxynitrite generation, and protein nitrotyrosylation in HM cells

**Figure 1:**
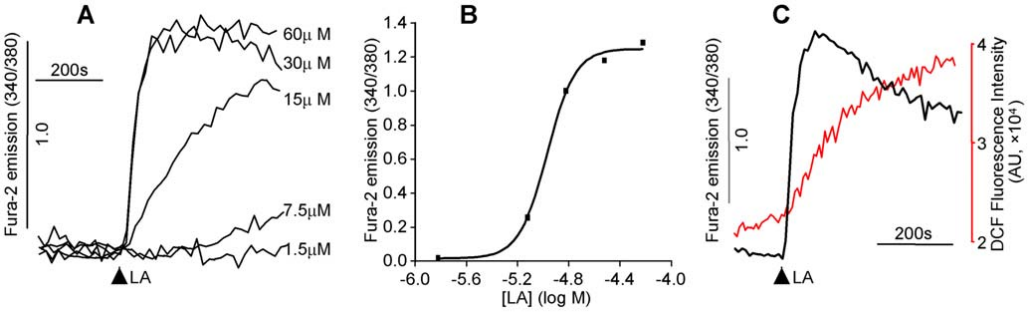
LA-induced [Ca^2+^]_i_ mobilization and ROS generation. Human mesangial cells were labeled with fura-2 (1 µM) alone or together with DCF (1 µM) (37°C, 30 min). The fluorescent intensity in the figures was presented as arbitrary unit (AU). (A): Traces represent the [Ca^2+^]_i_ responses induced by different concentrations of LA. (B): The curve shows the relationship between the amplitude of [Ca^2+^]_i_ responses and the LA concentrations. (C): Traces represent LA (30 µM)-induced [Ca^2+^]_i_ mobilization (black trace) and the associated ROS generation (red trace).

PIMCE and the indispensable role of hsp90β1 in the process have previously been described in the human teratocarcinoma NT2 cells [17]. To show that PIMCE is a contributing factor to diabetic nephropathy, we determined whether PIMCE occurs in primary cultured HM cells. As demonstrated in Figure 1, LA caused [Ca^2+^]_i_ rise in HM cells and the amplitude of the LA responses was dose dependent (Figure 1A and B) with maximal response observed at 30–60 µM. Thus 30 µM of LA was used in all subsequent experiments of this study. Furthermore, the [Ca^2+^]_i_ response of LA (30 µM) was associated with ROS generation in HM cells (Figure 1C).

**Figure 2 pone-0006048-g002:**
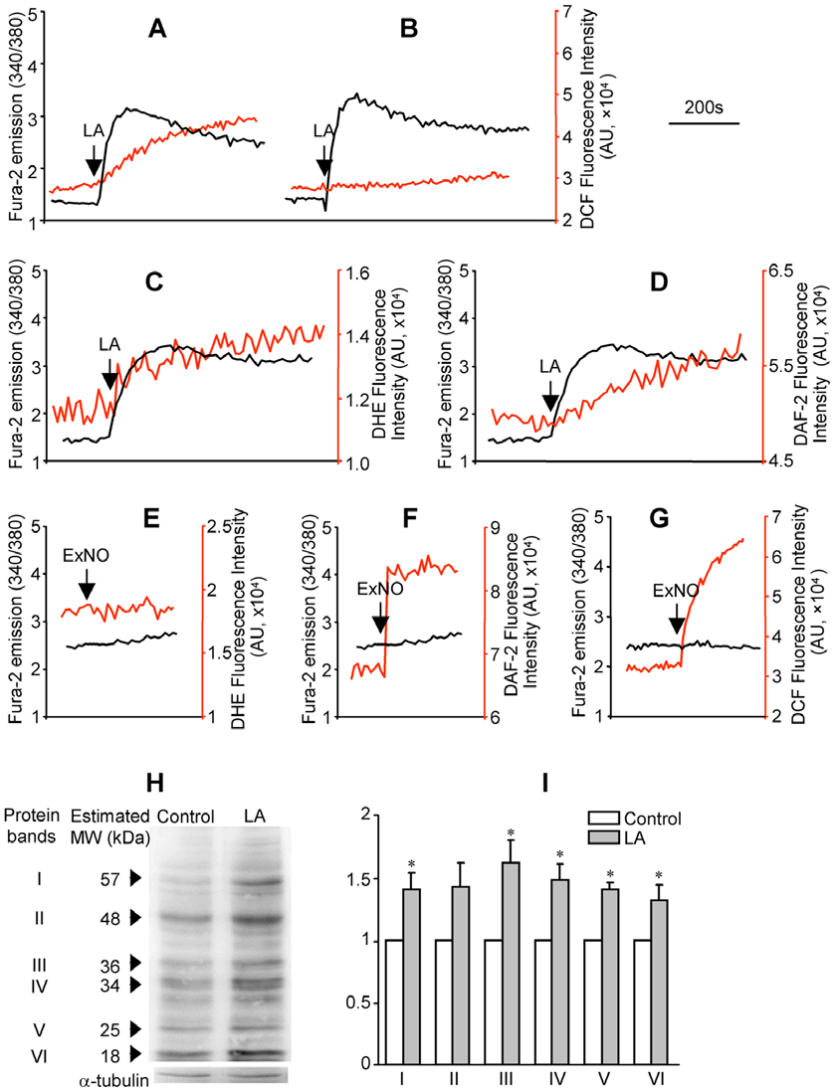
The LA-induced ROS signal was identified as peroxynitrite. (A): HM cells were labeled with fura-2 and DCF and traces represent LA-induced [Ca^2+^]_i_ mobilization (black trace) and ROS generation (red trace); (B): same measurements performed in cells treated with FeTPPS (a specific peroxynitrite decomposer; 0.5 µM, 30 min) prior to LA addition and the treatment inhibited the ROS signal significantly; (C) and (E): HM cells were labeled with fura-2 and dihydroethidium (DHE, 1 µM) and traces represent LA (C) or S-nitroso-N-acetylpenilillamine (exogenous NO donor, ExNO, 50 µM; E) responsive [Ca^2+^]_i_ mobilization (black trace) and superoxide generation (red trace); (D) and (F): HM cells were labeled with fura-2 and 4,5-diaminofluorescein diacetate (DAF-2, 0.5 nM) and traces represent LA (D) or ExNO (F) responsive [Ca^2+^]_i_ mobilization (black trace) and NO generation (red trace); (G) HM cells were labeled with fura-2 and DCF and traces represent ExNO responses in [Ca^2+^]_i_ (black trace) and peroxynitrite (red trace). The nitrotyrosine level in the cellular proteins was determined by western blot analysis in vehicle-(control) or LA-treated HM cells using α-tubulin as the loading control. The image (H) shows a representative experiment. The graphs (I) represent the mean ratio value±SEM of the density of protein band over α-tubulin. *P<0.05, n = 4, LA *vs* control.

Because nitrosative injury as indexed by protein nitrotyrosine was dramatically increased in diabetic nephropathy [11], we wanted to know whether the ROS generated along with LA-induced [Ca^2+^]_i_ mobilization in HM cells was peroxynitrite. Among different types of ROS, 2′,7′-dichlorodihydrofluorescein diacetate (DCF) preferentially reacts with peroxynitrite [18]. To determine the ROS signal observed in Figure 1C (as indexed by the DCF fluorescent intensity) was peroxynitrite, HM cells were treated with 5,10,15,20-Tetrakis(4-sulfonatophenyl) prophyrinato iron (III) (FeTPPS, 0.5 µM), a specific peroxynitrite decomposition catalyst [9], [19], [20]. Compared to control, pretreatment with FeTPPS did not affect the LA-induced [Ca^2+^]_i_ rise, but significantly attenuated the ROS production (Figure 2A and B; FeTPPS reduced the LA responsive DCF signal to 14.5±9.2% of control, *P*<0.001, n = 6), suggesting that the generated ROS were predominantly peroxynitrite. The data also indicated that the LA responsive peroxynitrite generation was either unrelated or occurred downstream of the [Ca^2+^]_i_ rise.

Since peroxynitrite is formed by the interaction of superoxide and NO, we next measured the generation of the latter two substances along with [Ca^2+^]_i_ mobilization by measuring dihydroethidium (DHE, a fluorescent indicator of superoxide), and diaminofluorescein-2 diacetate (DAF-2, an indicator of NO) fluorescence, respectively. As shown in Figure 2C and D, LA-induced [Ca^2+^]_i_ mobilization was associated with the generation of superoxide and NO. The specificity of DHE, DAF-2, and DCF was further demonstrated by using the exogenous NO donor (S-nitroso-N-acetylpenicillamine, 50 µM). The NO donor had no effect on DHE fluorescence (Figure 2E) but rapidly increased the fluorescent intensity of DAF-2 (Figure 2F). The NO donor also gradually increased DCF fluorescence, indicating the formation of peroxynitrite (Figure 2G).

Peroxynitrite is a strongly reactive oxidant that may interact with multiple proteins and cause nitrosative modification of tyrosine residues to form nitrotyrosine. We next measured the level of nitrotyrosine in cellular proteins of HM cells following LA treatment (30 µM, 1 h at 37°C). As shown in Figure 2H and I, LA treatment caused significantly increased nitrotyrosine levels in a spectrum of proteins in HM cells. The MW of these proteins ranged from 18 to 54 kDa (Figure 2H). These results further supported that the LA-induced [Ca^2+^]_i_ mobilization was associated with peroxynitrite generation and the latter leading to cellular protein nitrotyrosylation in HM cells.

### LA-induced mitochondrial Ca^2+^ efflux caused peroxynitrite production

**Figure 3 pone-0006048-g003:**
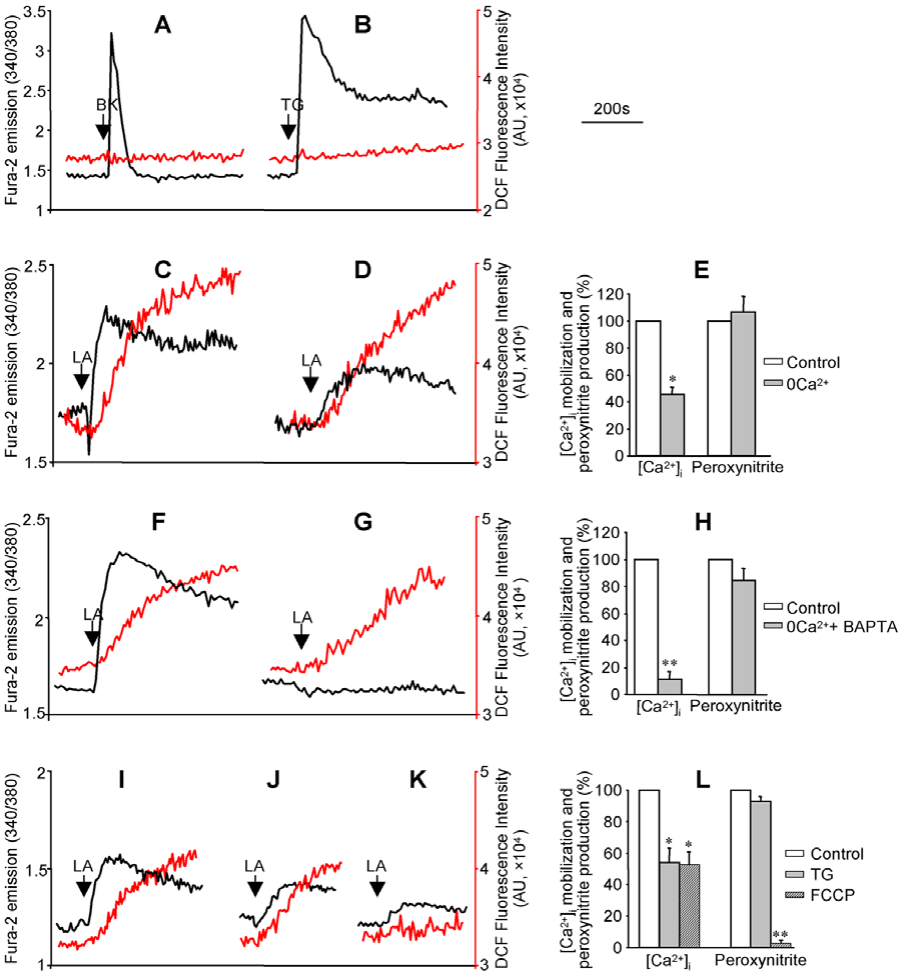
Mitochondria played an essential role in LA-induced [Ca^2+^]_i_ mobilization and peroxynitrite generation. HM cells were labeled with fura-2 and DCF to measure [Ca^2+^]_i_ mobilization (black traces) and peroxynitrite generation (red traces). (A): Bradykinin (BK, 100 nM) responses; (B): thapsigargin (TG, 2 µM) responses; (C) and (F): LA responses measured in the presence of extracellular Ca^2+^ (control); (D): LA responses measured without extracellular Ca^2+^; (G) LA responses measured in cells pretreated with BAPTA (50 µM, 30 min) without extracellular Ca^2+^. In the absence of extracellular Ca^2+^, (I): LA responses (control); (J): LA responses in cells pretreated with TG (2 µM, 5 min); (K) LA responses in cells pretreated with FCCP (a mitochondrial uncoupler; 4 µM, 5 min). The values in graphs (E, H, and L) represent the mean±SEM of the relative amplitudes of LA-induced [Ca^2+^]_i_ and peroxynitrite responses, *P<0.05, **P<0.01, n = 6, treated cells *vs* control.

Bradykinin (BK) and thapsigargin (TG) are known to induce [Ca^2+^]_i_ mobilization by activation of Ca^2+^ release from the ER and store-operated Ca^2+^ influx through inositol-1, 4, 5-trisphosphate dependent and independent mechanisms, respectively. However, as shown in Figure 3A and B, the [Ca^2+^]_i_ mobilization induced by both BK and TG was not associated with any detectable peroxynitrite production. These results indicated that the rise of [Ca^2+^]_i_ from the endoplasmic reticulum (ER) Ca^2+^ release and store-operated Ca^2+^ influx did not lead to peroxynitrite generation.

Even though LA-induced [Ca^2+^]_i_ mobilization was observed in the absence of extracellular Ca^2+^, chelation of extracellular Ca^2+^ significantly diminished LA-induced [Ca^2+^]_i_ signal (Figure 3C and D), demonstrating that both Ca^2+^ release from the internal Ca^2+^ stores and Ca^2+^ influx were involved in LA responsive [Ca^2+^]_i_ mobilization. The peroxynitrite responses to LA were unaffected by extracellular Ca^2+^ (Figure 3C–E). Moreover, clamping the [Ca^2+^]_i_ with 1,2-Bis(2-aminophenoxy)ethane-N,N,N',N'-tetraacetic acid (BAPTA, a membrane permeable chelator of cytosolic Ca^2+^) in the absence of extracellular Ca^2+^, abolished LA-induced [Ca^2+^]_i_ signal but had no significant effect on peroxynitrite generation (Figure 3F–H; *P*>0.05, n = 6), indicating that LA responsive peroxynitrite generation occurred independently of [Ca^2+^]_i_ rise. Because BAPTA does not prevent Ca^2+^ release from the internal Ca^2+^ stores, including ER and mitochondria, these experiments did not exclude the role of Ca^2+^ release from the internal Ca^2+^ stores in peroxynitrite generation.

Pretreatment of the cells with TG (2 µM) in the absence of extracellular Ca^2+^ to completely deplete the ER Ca^2+^ stores (as indicated by a complete inhibition of the BK-induced [Ca^2+^]_i_ signal following TG treatment, data not shown) only partially reduced the LA-induced [Ca^2+^]_i_ mobilization and had no significant effect on peroxynitrite generation (Figure 3I, J, L). These results indicated that additional Ca^2+^ stores other than ER, such as mitochondria, may be involved in LA-induced [Ca^2+^]_i_ response.

Interestingly, in the absence of extracellular Ca^2+^, pretreatment of the cells with carbonyl cyanide-p-trifluoromethoxyphenylhydrazone (FCCP, 4 µM), a well-known mitochondrial uncoupler, completely inhibited LA-induced peroxynitrite production and also attenuated the [Ca^2+^]_i_ response (Figure 3I, K, L). FCCP may alleviate LA-induced [Ca^2+^]_i_ by collapse of the mitochondrial proton gradient, which prevents Ca^2+^ uptake to the mitochondria and thus reduces the subsequent LA-responsive [Ca^2+^]_m_ efflux. These data indicated that at least part of the [Ca^2+^]_i_ response to LA resulted from [Ca^2+^]_m_ efflux, which might be essential to the peroxynitrite generation in HM cells.

**Figure 4:**
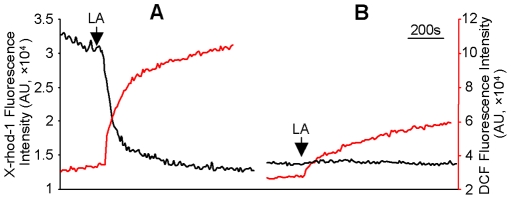
LA-induced mitochondrial Ca^2+^ efflux was responsible for peroxynitrite generation. Mitochondria were prepared from HM cells and labeled with X-rhod-1 (2 µM) and DCF (1 µM) with (A) or without Ca^2+^ (B). LA-induced mitochondrial Ca^2+^ efflux (black traces) and peroxynitrite generation (red traces) were indexed by the gradual decrease in X-rhod-1 and increase in DCF fluorescence intensity, respectively.

LA had been shown to increase [Ca^2+^]_i_ by causing [Ca^2+^]_m_ efflux in human teratocarcinoma NT2 cells [17]. We thus evaluated the role of LA-induced [Ca^2+^]_m_ efflux in peroxynitrite generation in isolated mitochondria from HM cells. Isolated mitochondria were labeled with X-rhod-1 and DCF to measure Ca^2+^ efflux and peroxynitrite generation, respectively. As shown in Figure 4A, LA caused Ca^2+^ efflux from the mitochondria as indexed by the reduced X-rhod-1 fluorescence intensity (black trace, Figure 4A). Interestingly, a robust increase in peroxynitrite generation was observed along with the [Ca^2+^]_m_ efflux, as indexed by the enhanced DCF fluorescence intensity (red trace, Figure 4A). Furthermore, chelation of Ca^2+^ to prevent mitochondria from Ca^2+^ refilling during X-rhod-1 and DCF labeling not only blocked LA-induced Ca^2+^ efflux from mitochondria (black trace, Figure 4B) but also significantly inhibited LA-induced peroxynitrite generation (red trace, Figure 4B), indicating that peroxynitrite generation was dependent on LA-induced [Ca^2+^]_m_ efflux.

### Hsp90β1 played a critical role in LA-induced mitochondrial Ca^2+^ efflux and peroxynitrite generation

**Figure 5:**
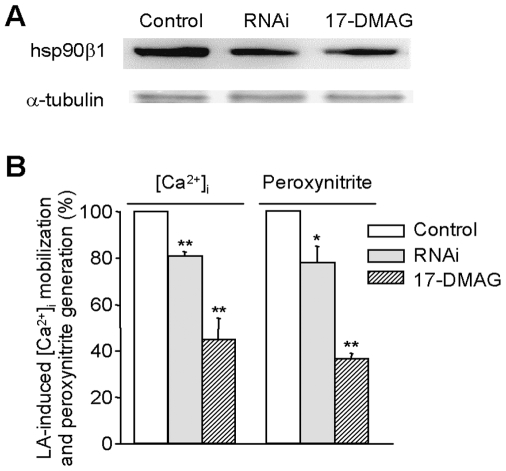
The effect of hsp90β1 on LA-induced [Ca^2+^]_i_ mobilization and peroxynitrite generation. HM cells were treated with vehicle (control), hsp90β1 RNAi, or 17-DMAG for 48 h. (A): Cell lysates were prepared and the alterations of hsp90β1 expression were analyzed by western blot analysis using α-tubulin as loading control. (B): The alteration of LA-induced [Ca^2+^]_i_ mobilization and peroxynitrite generation in hsp90β1 RNAi- and 17-DMAG-treated HM cells relative to control. The values represent the mean±SEM. *P<0.05, **P<0.01, n = 6.

Based on the indispensible role of hsp90β1 in PIMCE [17], it is expected that hsp90β1 would be essential for LA-induced peroxynitrite generation if the peroxynitrite response of LA indeed depends on [Ca^2+^]_m_ efflux in HM cells. We tested the role of hsp90β1 in the LA responses by downregulation of hsp90β1 expression with a well-known hsp90 chaperone inhibitor, 17-(dimethylaminoethylamino)-17-demethoxygeldanamycin (17-DMAG), and hsp90β1 RNAi. As shown in Figure 5A, treatment of HM cells with 17-DMAG (90 nM) or the hsp90β1 RNAi for 48 h effectively downregulated hsp90β1 expression. Of particular note, both treatments not only significantly inhibited LA-induced [Ca^2+^]_m_ efflux (as indicated by the alleviated [Ca^2+^]_i_ response) but also attenuated peroxynitrite generation (Figure. 5B). These results indicate that hsp90β1 is critical to LA-responsive [Ca^2+^]_m_ efflux and the coupled peroxynitrite responses.

### Mitochondrial complexes I and III were responsible for LA-induced peroxynitrite generation

**Figure 6:**
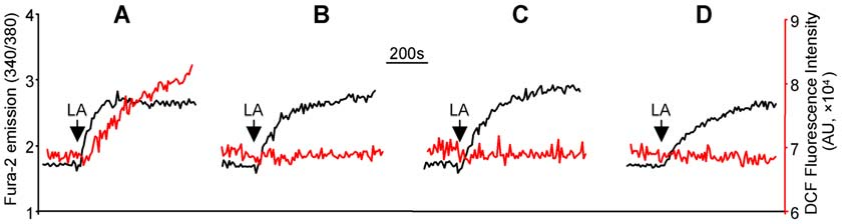
Mitochondrial complexes I and III were required for LA-induced peroxynitrite generation. HM cells were labeled with fura-2 and DCF to measure LA responsive [Ca^2+^]_i_ mobilization (black traces) and peroxynitrite generation (red traces). (A): LA responses in untreated cells (control); (B) LA responses in cells pretreated with rotenone (a mitochondrial complex I inhibitor, 10 µM, 20 min); (C) and (D): LA responses in cells pretreated with the mitochondrial complex III blockers, antimycin A (1 µM, 20 min; C) or myxothiazol (0.5 µM, 60 min; D).

It has been known that in the mitochondria ROS are produced predominantly by complexes I and III [6]. We thus evaluated whether mitochondrial complexes I and III were responsible for the LA-induced peroxynitrite generation in HM cells. Pretreatment of HM cells with the complex I inhibitor rotenone (10 µM, 20 min) completely diminished peroxynitrite generation (Figure 6A and B; *P*<0.001, n = 6) without significant effect on LA-induced [Ca^2+^]_i_ mobilization (*P*>0.05, n = 6). Blocking complex III with either antimycin A (1 µM, 20 min) or myxothiazol (0.5 µM, 60 min) also did not alter the [Ca^2+^]_i_ responses (*P*>0.05, n = 6) to LA but inhibited peroxynitrite generation (Figure 6A, C and D; *P*<0.001, n = 6). These results indicate that the mitochondrial complexes I and III are responsible for LA-induced peroxynitrite generation.

### LA-induced mitochondrial Ca^2+^ efflux and protein nitrotyrosylation were enhanced in diabetic mouse kidney

**Figure 7:**
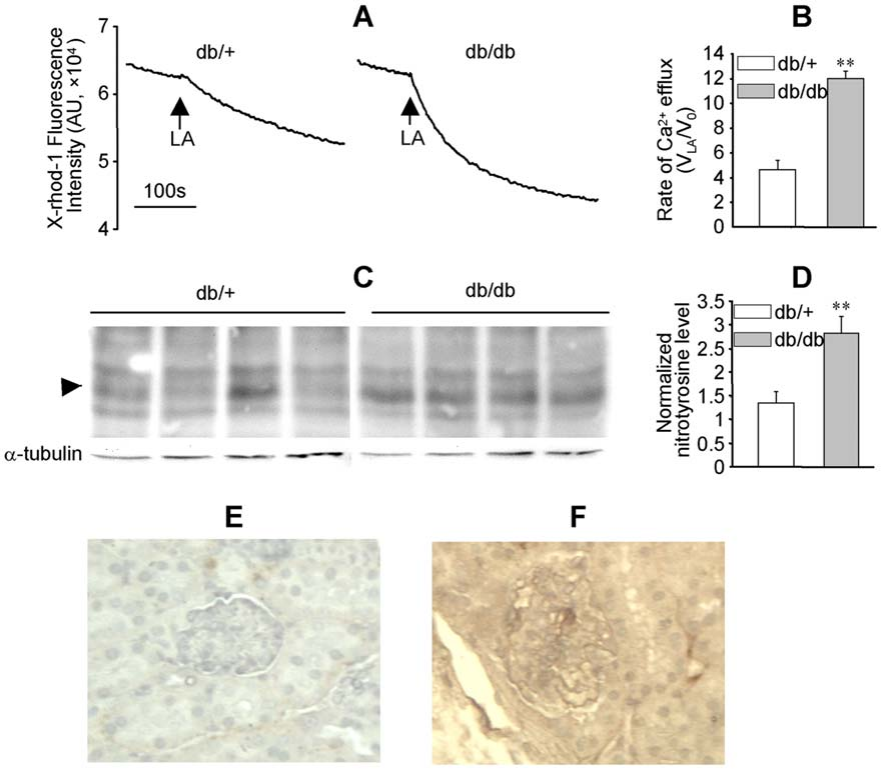
LA-induced mitochondrial Ca^2+^ efflux and protein nitrotyrosylation were enhanced in the kidney of db/db mice. Mitochondria were prepared from fresh kidney tissues of 12–16 wk old db/+ and db/db mice and labeled with X-rhod-1 (2 µM). (A): LA-induced mitochondrial Ca^2+^ efflux in the kidney of db/+ and db/db mice. (B): The ratio of the initial rate of LA-induced mitochondrial Ca^2+^ efflux (V_LA_; measured in the first 60 s following LA) to the basal rate (V_0_; measured prior to LA). The values represent mean±SEM of V_LA_/V_0_, **P<0.01, n = 3. (C) and (D): Nitrotyrosine levels in the kidney homogenates of four pairs of db/+ and db/db mice were analyzed by western blot using α-tubulin as loading control. The arrowhead highlights a protein band (∼22 kDa) with enhanced nitrotyrosine levels in db/db mice, **P<0.01, n = 4. (E) and (F): Representatives of the nitrotyrosine levels in the kidney sections of db/+ and db/db mice as determined by immunohistochemistry.

Functional and structural abnormalities were reported in the kidney of db/db mice, a type 2 diabetes model [21]. In mitochondria prepared from the kidneys of 12–16 wk old db/+ (littermate control) and db/db mice, LA caused Ca^2+^ efflux (Figure 7A and B). Compared to db/+ mice, the rate of LA-induced mitochondrial Ca^2+^ efflux was significantly enhanced more than two folds in db/db mice (Figure 7B, *P*<0.01, n = 3). Western blot analysis indicated that nitrotyrosylation in a protein with a MW about 22 kDa was significantly enhanced in db/db mice (Figure 7C and D, *P*<0.005, n = 4). Analysis of the nitrotyrosine level in kidney sections with immunohistochemistry further confirmed that protein nitrotyrosylation was enhanced in db/db mice kidney (Figure 7E and F).

## Discussion

A major role of [Ca^2+^]_m_ under physiological conditions is to stimulate oxidative phosphorylation by activation of multiple dehydrogenases [4] and ATP synthase [22] as well as regulation of other metabolic processes [6]. [Ca^2+^]_m_ may inhibit the generation of ROS from complexes I and III under normal conditions whereas its overload may promote ROS generation and apoptosis [6]. In our current work, we have demonstrated that LA (an 18∶2 n-6 PUFA) induced Ca^2+^ efflux from mitochondria and caused peroxynitrite production in HM cells. The LA responsive mitochondrial Ca^2+^ efflux may diminish the inhibitory effect of Ca^2+^ on ROS production from complexes I or III and thus leads to enhanced peroxynitrite generation (Figures 1–4, 6 and 8). These results revealed a cause and effect relationship between LA-induced [Ca^2+^]_m_ efflux and peroxynitrite production.

**Figure 8:**
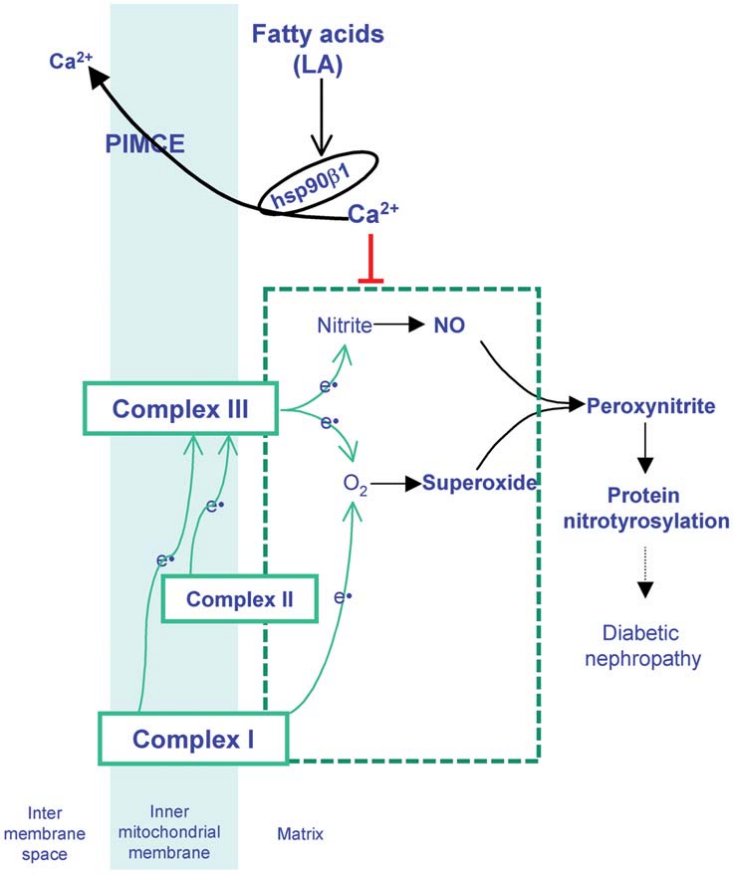
Mechanism of excessive NEFA contributing to pathogenesis of diabetic nephropathy. NEFA, such as linoleic acid (LA, an 18∶2 n-6 polyunsaturated fatty acid) induces Ca^2+^ efflux from mitochondria by activating a hsp90β1-dependent pathway (PIMCE). The LA responsive mitochondrial Ca^2+^ efflux diminishes the inhibitory effect of Ca^2+^ on superoxide production from complexes I or III and enhances nitrite conversion to NO by complex III, resulting in increased peroxynitrite formation and protein nitrotyrosylation. Protein nitrotyrosylation may disrupt the normal functions of mesangial cells and the kidney, leading to abnormalities in the structure and/or function of diabetic kidney.

Our observation uncovered a novel mechanism by which increased NEFA may contribute to the pathogenesis of diabetic nephropathy by increasing peroxynitrite generation. It has been shown that LA is a major NEFA component, and its concentration is significantly elevated in type 2 diabetic patients [23]. Elevated NEFA concentration has also been reported in the db/db mice and other diabetic animal models [24]. In the current work, we observed increased nitrotyrosylation in multiple proteins of LA-treated HM cells (Figure 2), demonstrating that the LA responsive peroxynitrite generation enhanced nitrosative stress. Increased nitrotyrosine levels were also observed in db/db mice along with enhanced LA responsive [Ca^2+^]_m_ efflux (Figure 7). Protein nitrotyrosylation may disrupt the normal functions of mesangial cells and the kidney, leading to abnormalities in the structure and/or function of diabetic kidney. Indeed, increased nitrotyrosine levels have been reported in the plasma and kidney of diabetic patients [25], [26].

Peroxynitrite is a highly reactive oxidant and is formed by the reaction between superoxide and NO. It has been reported that the mitochondrial complexes I and III cause superoxide production [7]. Previous studies described the existence of mitochondrial nitric oxide synthase (NOS), which produced NO in an L-arginine dependent manner [27], [28]. We have previously reported the expression of endothelial NOS (eNOS) and inducible NOS (iNOS), but not neuronal NOS, in rat mesangial cells [29]. The eNOS and iNOS expressed in mesangial cells may be responsible for NO productions induced by G protein coupled receptor agonists and inflammatory stimulators, respectively [29], [30]. The expression of iNOS in mesangial cells in response to inflammatory stimulators has been shown to be inhibited by conjugated-LA [30]. All three isoforms of NOS were detected in the kidney homogenates and the expression of NOS proteins was downregulated in aged ZSF1 fatty rats, which was associated with enhanced renal peroxynitrite and other structural and functional abnormalities [31]. The importance of NOS in the pathogenesis of diabetic nephropathy has clearly evidenced by studies from animal models and diabetic patients. However, in HM cells the LA-induced peroxynitrite generation was not inhibited by N^G^-nitro-L-arginine (0.1–1 mM) or L-N^G^-monomethyl arginine (0.5 mM) (data not shown), indicating that the LA responsive NO, a required substrate for peroxynitrite generation, was produced in an L-arginine independent manner. Mitochondrial complex III has been shown to possess nitrite reductase activity and is responsible for NO generation in the mitochondria by an L-arginine independent manner [32]–[34]. Thus the NO involved in LA responsive peroxynitrite generation was more likely to be produced from the nitrite conversion by mitochondrial complex III in HM cells (as depicted in Figure 8). The inhibition of LA responsive peroxynitrite generation by the complex I blocker (rotenone) or complex III blockers (antimycin A or myxothiazol) suggested that complexes I and III may be responsible for the generation of either superoxide or NO, which are the two primary substrates required to form peroxynitrite.

Our previous works had indicated that hsp90β1 played an important role in PIMCE [17]. In the current study, we showed that LA-induced [Ca^2+^]_m_ efflux and peroxynitrite generation were inhibited by 17-DMAG and, more specifically, hsp90β1 RNAi, indicating the hsp90β1 involvement (Figure 5). Based on the observations on the essential role of hsp90β1 in PIMCE, it is likely that hsp90β1 is involved in LA-induced peroxynitrite generation via regulation of [Ca^2+^]_m_ efflux.

In addition to diabetes, other pathological conditions have been shown to cause cellular NEFA accumulations. For example, excessive NEFA have been shown to accumulate in the kidney proximal tubules and cardiovascular tissues during ischemia-reperfusion [35], [36] and was responsible for sustained mitochondrial damage. The responses demonstrated in the current work (i.e. activation of [Ca^2+^]_m_ efflux and generation of peroxynitrite, Figure 1–4, and 6) may partially explain the deleterious effects of NEFA under a variety of disease conditions.

In summary, our work demonstrated that LA-induced [Ca^2+^]_m_ efflux caused peroxynitrite generation and led to increased nitrotyrosine in proteins. The enhanced nitrosative injury in response to LA may disrupt mesangial cell function in the kidney and thus contribute to the pathogenesis of diabetic nephropathy.

## Materials and Methods

### Cell culture

Primary human mesangial (HM) cells (a gift of Dr. H. E. Abboud, Department of Medicine, University of Texas Health Science Center at San Antonio) were prepared and cultured as previously described [37].

### Measurement of [Ca^2+^]_i_ and superoxide, NO, or peroxynitrite in HM cells

HM cells grown in 100 mm dishes were labeled with fura-2 (1 µM) alone to measure [Ca^2+^]_i_ or together with DHE (1 µM), DAF-2 (0.5 nM), or DCF (1 µM) as needed at 37°C for 30 min in RPMI 1640 medium to measure [Ca^2+^]_i_ and superoxide, NO, or peroxynitrite simultaneously. Loaded cells were harvested and resuspended in PBS1Ca buffer (containing 1 mM KH_2_PO_4_, 3 mM Na_2_HPO_4_, 154 mM NaCl, 1 mM CaCl_2_, 1 mM MgSO_4_, pH 7.4). [Ca^2+^]_i_ mobilization and superoxide, NO or peroxynitrite generation were monitored in a fluorometer (QM-6, Photon Technology International, NJ) using a cuvette with the temperature stabilized at room temperature. The ratio of fluorescence excited at 340 and 380 nm with emission of 510 nm was recorded and used to index the [Ca^2+^]_i_ change as previously reported [17]. With the multidye mode of the QM-6 fluorometer, fluorescence excited at 473, 488, or 506 nm and emitted at 595, 515, or 529 nm was measured to index the generation of superoxide, NO, or peroxynitrite in HM cells. The cell density was 1–2×10^6^ cells/ml in these experiments. The interference of LA to DHE, DAF-2, and DCF fluorescence was calibrated by subtracting the signal resulted from palmitic acid, which does not cause [Ca^2+^]_i_ mobilization in HM cells.

In experiments in which [Ca^2+^]_i_ mobilization was measured in the absence of extracellular Ca^2+^, the loaded cells were washed and measured in PBS0Ca buffer (containing 200 µM EGTA instead of 1 mM CaCl_2_ as in PBS1Ca).

### Measurement of [Ca^2+^]_m_ and peroxynitrite in mitochondria

Mitochondria were prepared from HM cells or the kidney of 12–16 wk old db/+ or db/db mice as previously described [17] and were suspended in MB1 buffer (containing 250 mM mannitol, 75 mM succinic acid, 0.1 mM EDTA, 0.5 mM EGTA, 10 mM HEPES, pH 7.4). The samples were diluted with nine volumes of HKG buffer (20 mM NaCl, 100 mM KCl, 1 mM CaCl_2_, 1 mM MgCl_2_, 10 mM pyruvate, 20 mM HEPES, pH 7.4) [17], and labeled with X-rhod-1 (2 µM) and DCF (1 µM) at 37°C for 30 min. Measurement with loaded mitochondria were performed in PBS0Ca buffer. Alteration of [Ca^2+^]_m_ and peroxynitrite generation were measured with fluorescence excited at 578 or 506 nm and emitted at 602 or 529 nm in a PTI QM-6 fluorometer, respectively. In these experiments, mitochondrial preparations equal to 40 µg/ml proteins were used.

In experiments in which LA-induced [Ca^2+^]_m_ efflux and peroxynitrite generation were determined in the absence of Ca^2+^ refilling, the mitochondrial preparations were suspended in MB1 buffer and diluted with nine volumes of HKG-EGTA buffer (containing 200 µM EGTA instead of 1 mM CaCl_2_ as in HKG), then labeled with X-rhod-1 and DCF and measured as described above.

### Downregulation of hsp90β1

HM cells in 100 mm dishes were grown for 24 h in RPMI 1640 supplemented with 14% FBS prior to treatment with hsp90β1 RNAi or 17-DMAG. The three hsp90β1 RNAi sequences used were: 1). UUAGCAAGACGUGUUCGAUUCGAGU; 2). UUCUGCUGGACCCAGCCAUG AAGUA; 3).UCAAACUGAGGCGAAGCAUUCUUUC (Invitrogen). Cells were treated with vehicle (Lipofectamine RNAi Max alone), a mixture of hsp90β1 RNAi (20 µM) and Lipofectamine-RNAi-Max, or 17-DMAG (90 nM) as described previously [17]. The cells were cultured for an additional 48 h and the effects of these treatments on hsp90β1 expression were verified by western blot analysis as described [17].

The effects of 17-DMAG and hsp90β1 RNAi on LA-induced [Ca^2+^]_i_ mobilization and peroxynitrite generation in treated HM cells were assessed as described above.

### Western blot analysis of nitrotyrosine

HM cells in 100 mm dishes were treated with LA (30 µM, 60 min at 37°C), lysed in ice-cold RIPA buffer (containing Tris 50 mM, NaCl 150 mM, NP-40 1%, Na-deoxycholate 0.25%, EDTA 1 mM, PMSF 1 mM, aprotinin1 µg/ml, leupeptin 1 µg/ml, pepstatin 1 µg/ml, Na_3_VO_4_ 1 mM, NaF 1 mM, pH 7.4), and kept on ice for 60 min. The mixture was centrifuged at 12,000×g for 15 min at 4°C. Western blot analysis of the proteins in the supernatant was performed with mouse mAb to nitrotyrosine (1∶1000, Upstate, Lake Placid, NY) and α-tubulin was used to control protein loading.

The kidneys of 12–16 wk old db/+ and db/db mice were collected, and cleaned slices (10–25 mg) of kidney tissues were homogenized in 1 ml RIPA buffer with a glass-glass homogenizer manually for 30 strokes. The homogenates were centrifuged at 150×g for 15 min at 4°C and the supernatants were collected. The nitrotyrosine levels were determined by western blot analysis.

### Immunohistochemistry analysis of nitrotyrosine in kidney sections

The kidneys of 12–16 wk old db/+ and db/db mice were fixed in formalin, embedded in paraffin and sectioned (4 µm). Paraffin sections were deparaffinized with xylene and hydrated with a series of graded ethanol washes. Endogenous peroxidase was inactivated by incubation for 30 min in 0.3% H_2_O_2_. The mouse mAb to nitrotyrosine was added for 1 h at room temperature. Tissue sections were treated with VECTASTAIN® ABC-Peroxidase Kit (Vector Laboratories, Burlingame, CA). The DAB Substrate Kit (Vector Laboratories) was used as the enzyme substrate. Sections were counterstained with CAT Hematoxylin (Biocare Medical, Concord, CA). Procedures involving the use of mice were approved by the Institutional Animal Care and Use Committee, University of Texas Health Science Center at San Antonio (0707-001).

### Statistics

Results are mean±SEM. Experimental data were analyzed using a two-tailed Student's t test for comparisons between two groups. A significant difference was defined at *P*<0.05.
